# Long term response stability of a well-type ionization chamber used in calibration of high dose rate brachytherapy sources

**DOI:** 10.4103/0971-6203.62200

**Published:** 2010

**Authors:** S. Vandana, S. D. Sharma

**Affiliations:** Radiological Physics and Advisory Division, Bhabha Atomic Research Centre, CT&CRS Building, Anushaktinagar, Mumbai - 400 094, India

**Keywords:** Brachytherapy, response stability, source calibration, well chamber

## Abstract

Well-type ionization chamber is often used to measure strength of brachytherapy sources. This study aims to check long term response stability of High Dose Rate (HDR)-1000 Plus well-type ionization chamber in terms of reference air kerma rate (RAKR) of a reference ^137^Cs brachytherapy source and recommend an optimum frequency of recalibration. An HDR-1000 Plus well-type ionization chamber, a reference ^137^Cs brachytherapy source (CDCSJ5), and a MAX-4000 electrometer were used in this study. The HDR-1000 Plus well-type chamber was calibrated in terms of reference air kerma rate by the Standards Laboratory of the International Atomic Energy Agency (IAEA), Vienna. The response of the chamber was verified at regular intervals over a period of eight years using the reference ^137^Cs source. All required correction factors were applied in the calculation of the RAKR of the ^137^Cs source. This study reveals that the response of the HDR-1000 Plus well-type chamber was well within ±0.5% for about three years after calibration/recalibration. However, it shows deviations larger than ±0.5% after three years of calibration/recalibration and the maximum variation in response of the chamber during an eight year period was 1.71%. The optimum frequency of recalibration of a high dose rate well-type chamber should be three years.

## Introduction

Brachytherapy is a mode of cancer treatment. The dose delivery to the tumor volume should be within ±5% in brachytherapy. Accurate delivery of doses to tumor in brachytherapy depends on many parameters. The accurate knowledge of the strength of the radioactive source is one of the most important parameters of brachytherapy treatment.[1] In the past there had been a practice to accept the manufacturer's quoted source strength without verification for the treatment planning.[1,2] Vendors often quote their source strength with large uncertainties of ±10%, which may lead to a large error in the dose delivery.[2] Errors from these types of practices have been demonstrated with measurements done at the National Institute of Standards and Technology (NIST), USA, using standards available at their laboratory.[1] Such type of errors can be avoided using properly calibrated dosimetry systems. For the calibration of brachytherapy sources, a well-type ionization chamber is recommended due to its advantage of having a large volume, reproducible geometry and nearly 4Π geometry when the source is at the position of maximum response.[1]

In brachytherapy, low as well as high activity sources are used. The HDR-1000 Plus well-type ionization chamber was specially designed for use with both high and low dose rate brachytherapy sources, using their respective calibration factors. Reference air kerma rate (RAKR) and air kerma strength (AKS) are the two internationally recommended quantities for the specification of brachytherapy source strength. RAKR and AKS are numerically equal but have different units. RAKR, commonly used for specification of the brachytherapy sources by many European and international bodies, is defined as the kerma rate to air, in air, at a reference distance of one meter, corrected for air attenuation and scattering.[3–5] AKS is also commonly referred to by various international agencies and is defined as the product of the air kerma rate in free space at a measurement distance, *r*, from the source center along the perpendicular bisector and the square of the measurement distance.[6–8]

It is well known that the response of a dosimetry system changes with time, which may introduce errors in the planning calculations. To avoid this type of error, dosimetry systems need to be calibrated periodically. In some countries, it is not possible for the user to have their well-type chambers calibrated on a recommended frequency due to the local unavailability of a standard laboratory. This study was aimed to check the temporal response stability of a well-type chamber for a long time so as to recommend the optimum frequency of calibration. This paper reports the result of a study carried out on the stability of the HDR-1000 Plus well-type ionization chamber in terms of the RAKR of a reference ^137^Cs source (model CDCSJ5) over a period of eight years.

## Materials and Methods

The HDR-1000 Plus (Standard Imaging Inc. USA) well-type chamber is maintained and used as a reference chamber for the calibration of various brachytherapy sources in our department. Table 1 shows the physical parameters of the HDR-1000 Plus well-type chamber. This chamber has an outer diameter (OD) of 102 mm, height of 156 mm and an active volume of 245 cm^3^. The outer wall of the chamber is made of 20 mm thick aluminum, which is sufficient to reduce the scattered radiation. For each source there is a unique source holder to place the source in a reproducible geometry inside the well of the chamber. The chamber can be used to measure the source strength in the range of 37×10^4^ Bq (0.01 mCi) to 74×10^10^ Bq (20 Ci) and has a sensitivity of approximately 1.97×10^10^ nA/Bq (7.3 nA/Ci). The HDR-1000 Plus well-type chamber is air vented, which provides an extra benefit to eliminate the problems associated with gas leakage in the case of pressurized well-type ionization chambers.

**Table 1:** Physical parameters of HDR-1000 Plus well-type ionization chamber

*Characteristic parameter*	*Specification*
Active Volume	245 cm^3^
Outer Diameter	102 mm
Height	156 mm
Sensitivity	1.97×10^10^ nA/Bq (7.3 nA/Ci)
Range	37×10^4^ Bq to 74×10^10^ Bq
Wall Material	Aluminum
Wall thickness	20 mm

Tedgren *et al* studied the ratios of RAKR measured by user and the vendors on 13 different afterloading units for a long duration.[9] They concluded that establishing a mean ratio of RAKR values as measured by user and vendor and monitoring this as a function of time is an easy way for early detection of problems associated with measuring dosimetry systems. We have used a ^137^Cs source to monitor the stability of our HDR-1000 Plus well-type chamber.

**Figure 1:**
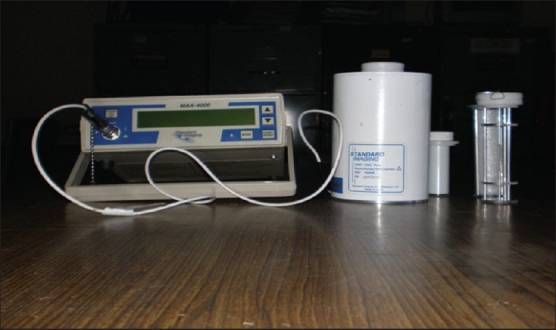
Photograph showing HDR-1000 Plus well-type ionization chamber, electrometer and source positioning jigs

A ^137^Cs source (total/active length = 20/13.5 mm, OD = 2.65 mm, model CDCSJ5, Nicomed-Amersham, Medi-Physics Inc., USA) was used as a reference source for checking the response stability of the HDR-1000 Plus well-type chamber. This reference ^137^Cs source (CDCSJ5) was calibrated locally using our HDR-1000 Plus well-type chamber which was calibrated at the Standards Laboratory of the International Atomic Energy Agency (IAEA) against their identical ^137^Cs source (CDCSJ5, total/active length = 20/13.5 mm, OD=2.65 mm). The ^137^Cs source (CDCSJ5) of IAEA had been calibrated in terms of reference air kerma rate at NIST, USA. The RAKR of our ^137^Cs source (CDCSJ5) was 201.539 *μ*Gy/hr as on March 23, 2000. Originally, HDR-1000 Plus well-type chamber of our department had been calibrated at Standards Laboratory of IAEA on October 4, 1999. The RAKR calibration factor was 5.040×10^11^ *μ*Gyh^−1^A^−1^ under standard environmental conditions (temperature 20°C, pressure 1013 mbar and relative humidity ≤50 %). This chamber was recalibrated at the Standards Laboratory of the IAEA on December 10, 2002 and a new RAKR calibration factor 5.011×10^11^ *μ*Gyh^−1^A^−1^ was determined under standard environmental conditions (temperature 20°C, pressure 1013 mbar and relative humidity ≤50 %). A MAX-4000 electrometer (Standard Imaging Inc., USA) was used along with the HDR-1000 Plus well-type chamber. This electrometer was calibrated separately and has a calibration factor of unity. This electrometer is very sensitive and has a very low leakage. It can be operated in rate as well as in integrated mode. Figure 1 is a photograph of the HDR-1000 Plus well-type ionization chamber, MAX-4000 electrometer and source positioning jigs.

Before starting the measurements, 30 minutes time was given to the whole system to achieve electronic and environmental stability. The well response of the HDR-1000 Plus well-type chamber using a reference ^137^Cs source (CDCSJ5) was measured. During the study, the chamber was kept in the middle of the room such that it was 1.5 meters away from all walls and at a height of one meter from the floor to avoid any room scattering contribution. To determine the position of maximum response, the source was placed in the source holder and moved upward from the bottom of the well in 5 mm steps. During these measurements, the electrometer was operated in integrated mode and readings were integrated for 120 seconds at each source position. The response stability check of the HDR-1000 Plus well-type chamber was performed on a quarterly basis (occasionally earlier or later) over a period of eight years. For this purpose, the reference ^137^Cs source (CDCSJ5) was kept at the maximum response position of the well and the charge collected for 180 seconds was recorded. The electrometer reading so obtained was corrected for background reading, charge leakage, source decay and variation in environmental conditions. The RAKR and percentage variation in response (PVR) were determined using the following equations

(1) $$RAKR=\left(MR\right)c*{\left(N_{RAKR}\right)}_{Cs-137,\;CDCSJ5}$$

(2) $$PVR=\frac{{\left(RAKR\right)}_{reference}-{\left(RAKR\right)}_{measured}}{{\left(RAKR\right)}_{reference}}*100$$

Where the RAKR = reference air kerma rate, (MR)_C_ = the meter-reading corrected for background, charge leakage and environmental conditions, and (N_RAKR_)_Cs-137, CDCSJ5_ = the RAKR calibration factor (*μ*Gyh^−1^A^−1^) of the well-type chamber for ^137^Cs source (CDCSJ5); (RAKR)_reference_ = decay corrected RAKR of the reference ^137^Cs source (CDCSJ5), and (RAKR)_measured_ = the RAKR of the reference ^137^Cs source (CDCSJ5) measured by HDR-1000 Plus well-type chamber.

## Results and Discussion

**Figure 2:**
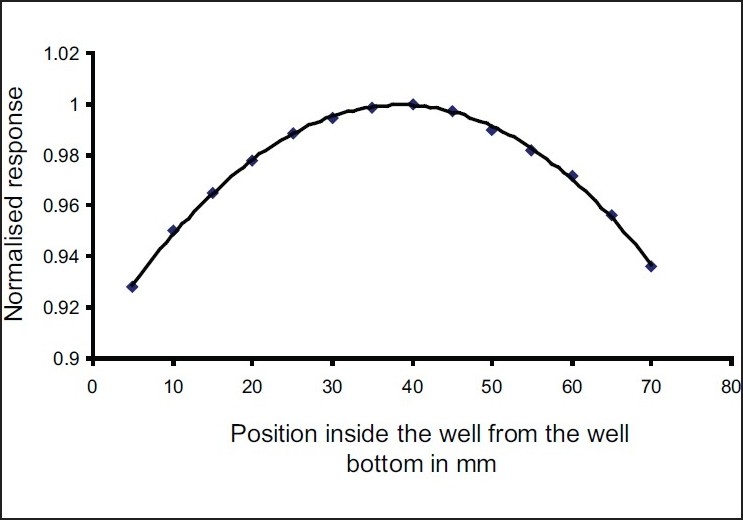
Response of HDR-1000 Plus well-type ionization chamber to a reference ^137^Cs source (CDCSJ5)

The well response curve of the chamber is shown in Figure 2. From this figure it can be seen that the maximum response of the HDR-1000 Plus for our CDCSJ5 ^137^Cs reference source is at a height of about 35 - 45 mm from the well bottom. The midpoint (40 mm) was chosen as source position for subsequent measurement of RAKR.

**Figure 3:**
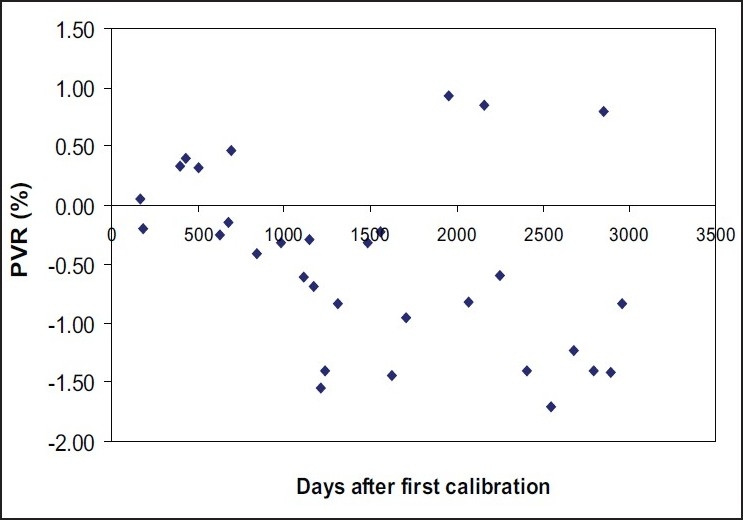
Temporal response of HDR-1000 Plus well-type ionization chamber to a reference ^137^Cs source (CDCSJ5) after first calibration

As per international requirements, the long term response stability of the well-type ionization chamber should be within ±0.5% over a period of two years as verified using a calibrated check source. The variation in response of the well chamber over a period of eight years is shown in Figure 3 where only the initial calibration factor (5.040×10^11^ *μ*Gyh^−1^A^−1^) of the chamber was taken into account (the recalibration factor was not used in this representation). Data of this figure show that the response of the chamber was well within ±0.5% for about 1100 days (about 3 years) after first calibration and then it started showing variation higher than ±0.5%. It can also be seen from this plot that though majority of the data are beyond the limit of ±0.5% after three years of the first calibration, a few data points are well within the acceptable limit of ±0.5%. This may be due to random variation in response of the dosimetry system or emission from impurities in the source such as ^134^Cs (<1%).

**Figure 4:**
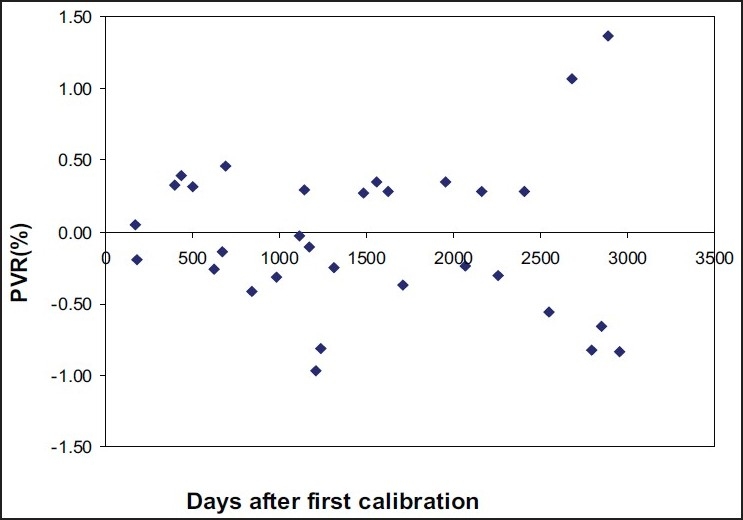
Temporal response of HDR-1000 Plus well-type ionization chamber to a reference ^137^Cs source (CDCSJ5) after first and second calibration

Figure 4 shows the time response graph for the eight year study duration, in which the first calibration factor (5.040×10^11^*μ*Gyh^−1^A^−1^) as well as second calibration (recalibration) factor (5.011×10^11^*μ*Gyh^−1^A^−1^) of the chamber were taken into account. The graph presents percentage variation of the measured RAKR from the calculated RAKR of the ^137^Cs reference source (Equation 2). For three years after the first calibration, use was made of the first calibration factor while determining the percentage variation in the measured RAKR value. For the period after three years of first calibration, the second calibration factor was used while determining the percentage variation in measured RAKR. This graph shows that the chamber response is stable within ±0.5% for three years after first calibration, and for next 3.5 years after the second calibration. It also shows that the variation in response is beyond ±0.5% after 3.5 years, even if the second calibration is applied. Though majority of the data shows variations within ±0.5%, a few data points show percentage variation in RAKR more than ±0.5%, which is assumed as the random fluctuation of the measuring system. The maximum variation observed was up to ±1.71% for the eight year study period.

As mentioned earlier, the HDR-1000 Plus well-type chamber was first calibrated on October 4, 1999 and recalibrated on December 10, 2002 at the Standards Laboratory of IAEA. The time interval between the two calibrations is more than three years. The variation of about 0.6% in these two calibration factor can be attributed to the time interval of more than three years. The two calibration factors were used independently in our studies. The first calibration factor was used to evaluate stability up to November 2002, and the second calibration factor was used to evaluate the stability of the HDR-1000 Plus well-type chamber after December 2002. However, the data shown in Figure 3 employed the first calibration factor only. Although the chamber was recalibrated after 1111 days, the second calibration factor was not used in the calculation so as to demonstrate the actual variation in stability of the chamber over a long duration. This presentation of the data simulates the condition in which a well-type chamber may not be calibrated for an extended time (> 3 years).

The internationally recommended frequency of calibration for radiotherapy dosimetry systems, including well-type ionization chambers, is two years (IAEA, 2002), but in our country it is three years. Though the study was carried out with a Standard Imaging HDR-1000 Plus well-type ionization chamber, results obtained here could also be applied to the PTW/Nucletron well-type ionization chamber (Source Dosimetry System) used for calibration of high dose-rate brachytherapy sources as sensitivity of this well-type chamber varies with time by a similar magnitude.[10]

## Conclusions

Long term response of the HDR-1000 Plus well-type chamber was monitored at regular intervals for a period of eight years using a CDCSJ5 ^137^Cs reference source. This study shows that the HDR-1000 Plus well-type ionization chamber response is very stable within the tolerance value of ±0.5 % over a period of 3 - 3.5 years after calibration, but it needs recalibration at an interval of three years because its response changes with time. As per international protocol, an optimum frequency of calibration for a dosimetry system is two years. However, 3 - 3.5 years can be acceptable as a frequency of calibration when a standards laboratory is not available in the country.
